# Marimastat as maintenance therapy for patients with advanced gastric cancer: a randomised trial

**DOI:** 10.1038/sj.bjc.6600310

**Published:** 2002-06-17

**Authors:** S R Bramhall, M T Hallissey, J Whiting, J Scholefield, G Tierney, R C Stuart, R E Hawkins, P McCulloch, T Maughan, P D Brown, M Baillet, J W L Fielding

**Affiliations:** Department of Surgery, Queen Elizabeth Hospital, Birmingham, UK; Department of Surgery, Queen's Medical Centre, Nottingham, UK; Department of Surgery, Glasgow Royal Infirmary, Glasgow, UK; CRC Department of Medical Oncology, Christie Hospital, Manchester, UK; Department of Surgery, University Hospital Aintree, Liverpool, UK; Department of Oncology, Velindre Hospital, Cardiff, UK; British Biotech Pharmaceuticals Ltd., Oxford, UK

**Keywords:** gastric cancer, marimastat, matrix metalloproteinase inhibitors

## Abstract

This randomised, double-blind, placebo-controlled study was designed to evaluate the ability of the orally administered matrix metalloproteinase inhibitor, marimastat, to prolong survival in patients with non-resectable gastric and gastro-oesophageal adenocarcinoma. Three hundred and sixty-nine patients with histological proof of adenocarcinoma, who had received no more than a single regimen of 5-fluorouracil-based chemotherapy, were randomised to receive either marimastat (10 mg b.d.) or placebo. Patients were treated for as long as was tolerable. The primary endpoint was overall survival with secondary endpoints of time to disease progression and quality of life. At the point of protocol-defined study completion (85% mortality in the placebo arm) there was a modest difference in survival in the intention-to-treat population in favour of marimastat (*P*=0.07 log-rank test, hazard ratio=1.23 (95% confidence interval 0.98–1.55)). This survival benefit was maintained over a further 2 years of follow-up (*P*=0.024, hazard ratio=1.27 (1.03–1.57)). The median survival was 138 days for placebo and 160 days for marimastat, with 2-year survival of 3% and 9% respectively. A significant survival benefit was identified at study completion in the pre-defined sub-group of 123 patients who had received prior chemotherapy (*P*=0.045, hazard ratio=1.53 (1.00–2.34)). This benefit increased with 2 years additional follow-up (*P*=0.006, hazard ratio=1.68 (1.16–2.44)), with 2-year survival of 5% and 18% respectively. Progression-free survival was also significantly longer for patients receiving marimastat compared to placebo (*P*=0.009, hazard ratio=1.32 (1.07–1.63)). Marimastat treatment was associated with the development of musculoskeletal pain and inflammation. Events of anaemia, abdominal pain, jaundice and weight loss were more common in the placebo arm. This is one of the first demonstrations of a therapeutic benefit for a matrix metalloproteinase inhibitor in cancer patients. The greatest benefit was observed in patients who had previously received chemotherapy. A further randomised study of marimastat in these patients is warranted.

*British Journal of Cancer* (2002) **86**, 1864–1870. doi:10.1038/sj.bjc.6600310 www.bjcancer.com

© 2002 Cancer Research UK

## 

Gastric cancer is the fourth most common cause of cancer death in Europe, with an incidence of 24 per 10^5^ population in males and 16 per 10^5^ population in females (Stadtlander and Waterbor, 1999). Even in the US, where there has been a sharp decline in the incidence of gastric cancer over the past 50 years, the incidence of adenocarcinoma of the gastro-esophageal junction has risen rapidly (Devesa *et al*, 1998).

Gastric cancer spreads by local extension to form lymphatic, peritoneal and distant metastases. As a result of early spread only 10–20% of patients present with resectable disease. For the remainder, surgical debulking will be attempted in some, while others will be considered inoperable. Until recently there has been relatively little evidence of a survival benefit for established cancer therapies in this disease. However, results from a recent US study have shown a significant survival benefit for the use of adjuvant 5-fluorouracil (5-FU) and radiation in patients following curative resection (MacDonald *et al*, 2001) and a meta-analysis of the available randomised evidence also supports the use of adjuvant chemotherapy following curative resection (Mari *et al*, 2000).

In patients with inoperable gastric cancer, the data regarding the use of chemotherapy are less clear. A survival benefit has been shown for one regimen *vs* another (Cirera *et al*, 1999; Roth *et al*, 1999; Waters *et al*, 1999; Chao *et al*, 2000) and in a small number of studies with small numbers of patients comparing chemotherapy with best supportive care (Murad *et al*, 1993; Pyrhonen *et al*, 1995; Glimelius *et al*, 1997). The absence of a large randomised study of chemotherapy *vs* best supportive care has allowed the debate on its merits to continue. As a result a wide range of 5-FU based chemotherapy regimens are offered to patients, and a proportion of patients are offered no cytotoxic therapy at all.

Recent changes in attitude towards the non-operative management of solid gastrointestinal tumours have led to a renewed interest in the development of novel agents. The rapid increase in knowledge of the molecular and cellular biology of malignancy over the last decade has enabled scientists to accurately target cellular pathways with synthetic compounds and inhibit these pathways for potential therapeutic benefit. Several of these strategies have been tested in clinical trials in patients with a variety of tumour types. One such treatment strategy has been the inhibition of matrix metalloproteinases (MMPs).

The MMPs are a family of proteolytic enzymes that are responsible for the breakdown of connective tissue proteins. These enzymes play an important role in normal processes of growth, differentiation and repair. The activity of MMPs is tightly regulated at several levels including gene expression and inhibition by tissue inhibitors known as TIMPs. There is now considerable evidence however, that aberrant MMP expression contributes to the invasive growth and spread of a variety of solid malignancies (Chambers and Matrisian, 1997). MMP-2 (gelatinase A), MMP-9 (gelatinase B) (Sier *et al*, 1996), MMP-7 (matrilysin) (Honda *et al*, 1996) and MMP-14 (MT1-MMP) (Nomura *et al*, 1995) are over-expressed in human gastric cancer. It is therefore feasible that specific MMP inhibitors might restore the normal balance of proteolytic activity and thereby prevent further tumour growth and metastasis.

Marimastat (BB-2516) is a broad spectrum, low molecular weight MMP inhibitor with IC_50_s against purified enzymes in the low nanomolar range (Whittaker *et al*, 1999). The closely related inhibitor batimastat (BB-94) has been shown to inhibit tumour growth and spread in a range of cancer models (Chirivi *et al*, 1994; Eccles *et al*, 1996) and marimastat has been shown to inhibit tumour growth in a xenograft model of human gastric cancer (Watson *et al*, 1999). MMP inhibitors have not been shown to cause tumour regression in cancer model studies and it was therefore proposed that these agents be tested in the clinic as oncostatic treatments.

Support for the current study came from the results of a phase I trial of marimastat in patients with advanced gastric cancer (Tierney *et al*, 1999). Patients in this pilot study received marimastat at doses of marimastat 25 mg o.d. or 50 mg b.d. An increase in endoscopically observed fibrous stroma, and a decrease in haemorrhagic appearance, were observed in approximately one third of patients. These changes were frequently accompanied by signs of improved clinical well-being. The dose of marimastat used in the current study (10 mg b.d.) was selected on the basis of series of phase II cancer antigen studies, which explored the biological activity and tolerability of the compound (Nemunaitis *et al*, 1998; Primrose *et al*, 1999). The principal treatment-related side effect recorded in these studies was musculoskeletal pain and inflammation, commonly in the shoulder girdle and joints of the hands. These side effects usually resolved rapidly on treatment interruption, however it was judged that the more rapid onset of musculoskeletal pain at doses above 10 mg b.d. would prohibit chronic administration.

The current multi-centre randomised study was designed to establish whether the encouraging biological activity seen in the phase I study would translate into a significant survival advantage for gastric cancer patients receiving marimastat.

## PATIENTS AND METHODS

### Patient population

Patients with histologically or cytologically proven adenocarcinoma of the stomach, which was locally advanced or metastatic, and was considered inoperable, were eligible for inclusion. Adenocarcinoma of the gastro-oesophageal junction was permitted if in the opinion of the investigator the tumour was primarily gastric in origin. If a non-curative surgical resection had been performed, patients were required to have confirmation of residual disease and to have entered the study within 4 weeks of surgery. Previous first-line 5-fluorouracil-based chemotherapy was allowed. Patients who received chemotherapy must have entered the study between 2 and 6 weeks following the last dose, and must not have shown clinical evidence of disease progression before entry. Patients undergoing open surgery within 2 weeks or laparoscopic surgery within 1 week of study entry; pregnant or lactating patients were also excluded.

Patients were required to be aged over 18 years and have an ECOG performance status of 0 or 1. Patients had to have adequate bone marrow reserve at study entry, (absolute granulocyte count of >0.5×10^9^/L and platelet count of >50×10^9^/L). Adequate baseline hepatic function (bilirubin<twice the upper limit of normal and aspartate transaminase (AST), alanine transaminase (ALT) <1.5 times the upper limit of normal) and adequate renal function (serum creatinine <1.5 times the upper limit of normal) were also required. Patients were excluded if they had received radiotherapy, more than one course of systemic anti-cancer therapy, any previous investigational agent, hormonal anti-neoplastic therapy, or prior exposure to a metalloproteinase inhibitor.

Signed and witnessed informed consent was obtained from each patient prior to study entry. The study was performed in accordance with the Declaration of Helsinki, approved by Institutional Review Boards and local regulatory authorities and conducted in accordance with the FDA Guideline on Good Clinical Practice.

### Patient assignment

Patients were assigned to marimastat (10 mg b.d.) or matched placebo in a ratio of 1 : 1 by means of a minimisation program using the following criteria: centre, gender, prior chemotherapy (yes/no), chemotherapy regimen, response to chemotherapy, performance status, extent of disease at baseline (local/metastatic), and prior non-curative surgery (yes/no). The minimisation program also contained a random element in the event of tied minimisation scores for assigning treatments (Taves, 1974).

### Treatment

Marimastat or matched placebo were administered in a double blind fashion as 10 mg capsules. If musculoskeletal toxicities developed, treatment was omitted until the symptoms had abated. Patients could then restart treatment at the same dose. If patients experienced a recurrence of their symptoms, treatment was again omitted until the symptoms had abated and treatment could then be restarted once daily (i.e. a 50% dose reduction). If the symptoms recurred again further treatment was at the physician's discretion, after consideration of the risk/benefit ratio for the individual patient. Once a dose reduction had been mandated, no escalation to the previous level was permitted. Patients on treatment were seen at 6 weeks, and then every 3 months, or at early termination for any reason. Treatment assignments were kept in sealed envelopes at British Biotech. The blind was only broken once all data had been verified, collected and quality assured, and the statistical analysis plan had been finalised and signed.

### Endpoints and statistical analyses

The primary study endpoint was overall survival in the intention-to-treat population. A secondary analysis of overall survival in the sub-group of patients who had received chemotherapy was also pre-defined in the statistical analytical plan, since this sub-group might differ in response or outcome from the chemonaive patients. Progression-free survival, quality of life and safety and tolerability were defined as secondary endpoints. The original sample size for this study was 150 per arm. This was increased to 180 per arm when it became apparent that a higher than expected proportion of patients were leaving the study due to early disease progression (within 8 weeks of entry). The revised sample size was estimated to be sufficient to show a statistically significant difference in survival between the marimastat and placebo groups, assuming population mortality rates at 18 months of 85% for placebo and 72% for marimastat (a 15% relative reduction *vs* placebo), with at least 90% power and using α=0.05 (two-tailed log rank test). The prospectively defined analysis point for the study was when 85% of either group had died or 18 months after the last patient was recruited, whichever occurred sooner. This point was reached in January 1999 with 85% mortality in the placebo arm. A further 2-years of survival follow-up have also been obtained up to January, 2001.

The treatment groups were compared using Kaplan-Meier survival curves and tested using the log-rank test. In all survival analyses, patients who were lost to follow up were censored at last known date alive. In addition, these results were supplemented with hazard ratios (HR) with a 95% confidence interval using a Cox proportional hazard model containing only treatment. Quality of life data were analysed using a Wilcoxon rank-sum test and proportions were analysed using a χ^2^ test.

### Efficacy and safety evaluation

The primary efficacy endpoint in this study was survival. Treatment continued until death, disease progression or drug toxicity that warranted removal from the study. Patients could receive salvage chemotherapy or other conventional anti-cancer therapy once they had withdrawn from the study.

Time to disease progression was defined as the time from minimisation to documented disease progression (clinical or radiological). Progressive disease was defined as a >25% increase in the sum of the products of the largest perpendicular diameters of all measurable lesions from the study nadir. If lesions were not bi-dimensional then an unequivocal worsening of any evaluable lesion as determined by more than one investigator, the appearance of new lesions or death would constitute progressive disease. CT scans were performed at baseline and thereafter at 3 monthly intervals or at early termination if there was clinical suspicion of relapse. Patients dying prior to documented progressive disease were considered to have experienced progressive disease at death. Quality of life was measured by the QLQ-C30 questionnaire at screening, weeks 6 and 12 and every 3 months thereafter up to 36 months.

Medical history was recorded at baseline. Performance status, full blood count and serum chemistry profile were recorded at baseline, after 6 weeks, 3 months and 3 monthly thereafter up to 18 months. All signs, symptoms and laboratory abnormalities were assessed using the NCI CTC criteria for toxicities. In addition, a specific rating for grading musculoskeletal toxicity was developed for this study. Grade 1 for musculoskeletal toxicity was defined as aches and pains with no restriction of activity. Grade 2 was defined as having pain causing restriction of activity. Grade 3 was defined as having pain and the presence of nodules or clinically inflamed joints or tendons. Grade 4 was defined as pain and the presence of a contracture.

## RESULTS

### Patient characteristics

A total of 369 patients were recruited from 37 European hospitals between October 1996 and October 1998. Of these, 185 received marimastat and 184 received placebo. One patient in the marimastat arm was lost to follow-up. The baseline patient demographics are shown in Table 1

**Table 1:**
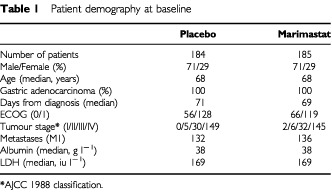
Patient demography at baseline

and the pre- and post-study study treatments are summarised in Table 2

**Table 2:**
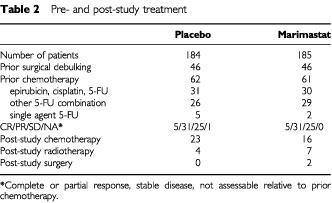
Pre- and post-study treatment

. The two treatment arms were well balanced for known prognostic variables. The majority of patients entered the study with advanced metastatic disease (80% stage IV, 73% M1). Overall, 25% of patients had undergone a partial gastrectomy or oesophagogastrectomy and one third of the patients had received chemotherapy prior to entering the study. A small proportion of patients were offered salvage chemotherapy after completing the marimastat study.

Seventeen patients were deemed to be protocol deviators, 10 in the marimastat group and seven in the placebo group. Two marimastat patients and one placebo patient had potentially curative resections and entered the study without histological or radiological confirmation of residual or relapsed disease. Two marimastat patients and one placebo patient had received prior radiotherapy. Previous malignant disease was recorded for one marimastat patient (testicular cancer) and two placebo patients (testicular and cervical cancer). Six marimastat patients and three placebo patients showed signs of disease progression between completion of first-line treatment and study entry, and did not take any study drug even though they were minimised. In addition, six marimastat patients and three placebo patients entered the study with disease that would normally be considered resectable (T1-2, N0-1). However, a combination of advanced age and overall frail condition precluded the possibility of resection. Although technically these patients did not deviate from the protocol they may be considered to have introduced an imbalance in the arms and this is explored in the following section.

Pharmacokinetic analysis revealed mean plasma concentrations of marimastat of 67.6 ng ml^−1^ at week 6 (*n*=147) and 49.2 ng ml^−1^ at week 12 (*n*=101). Due to variability in sampling times these values cannot be regarded as true troughs. The concentrations were in line with expectations from previous studies and equate to free-drug concentrations of approximately 3–4 times the IC_50_ for the target enzymes such as gelatinase A and MT1-MMP.

### Efficacy analyses

**Figure 1:**
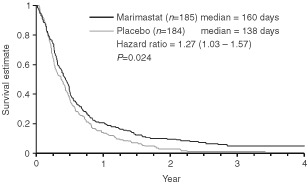
Overall survival (intention to treat).

The primary analysis was performed when 85% of patients in the placebo arm had died. The primary endpoint of the study was met when the nine patients who did not take the study drug were excluded (modified ITT) (*P*=0.043 log-rank test, HR=1.27 (1.00–1.60)), but failed to meet the endpoint when all patients are included (ITT) (*P*=0.07 log-rank test, HR=1.23 (0.98–1.55)). The survival difference in favour of marimastat was maintained with a further 2 years of follow-up (ITT, *P*=0.024 log-rank test, HR=1.27 (1.03–1.57)), with median survival times of 160 days for marimastat and 138 days for placebo, and 2-year survival of 9% and 3% respectively (Figure 1

). The modified ITT analysis showed a marginally greater survival benefit (*P*=0.014 log rank test, HR=1.30 (1.05–1.61) for result after 2 years of additional follow-up.

**Figure 2:**
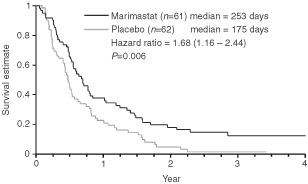
Overall survival (Chemotherapy sub-group).

Analysis of overall survival in the predefined sub-group of patients who had received prior chemotherapy revealed a significant benefit for marimastat at the primary analysis point (*P*=0.045 log-rank test, HR=1.53 (1.00–2.34)). This survival difference increased with 2 years of additional follow-up (*P*=0.006 log-rank test, HR=1.68 (1.16–2.44)), with median survival times of 253 days for marimastat and 175 days for placebo, and 2-year survival of 18% and 5% respectively (Figure 2

). Importantly, there was no evidence of an adverse effect on survival in the patients who had not received chemotherapy (*P*=0.515 log-rank test, HR=1.09 (0.84–1.40)).

**Figure 3:**
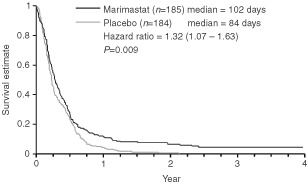
Progression-free survival (intention to treat).

There was also a significant benefit in progression-free survival at the primary analysis point (*P*=0.014 log-rank test, HR=1.31 (1.05–1.63)). This difference was maintained over the 2 years of additional follow-up (*P*=0.009 log-rank test, HR=1.32 (1.07–1.63)) (Figure 3

).

The possibility of bias or imbalance between the arms was explored as an explanation for the survival differences. Interactions between several factors precluded a single multifactorial analysis to adjust for baseline prognostic factors. There was a small imbalance in ECOG status in the ITT population with 70% of placebo patients having a status of 1 *vs* 64% of marimastat patients. Other baseline prognostic factors were well balanced for the ITT population. The chemotherapy sub-group was found to have minor imbalances in favour of marimastat for ECOG status (ECOG 1 57% *vs* 63% for marimastat and placebo respectively), and in favour of placebo for metastases (M1 72% *vs* 68%) and no prior resection (74% *vs* 65%). Another source of imbalance was recruitment of three placebo patients and six marimastat patients who had resectable disease (T1-2, N0-1). The chemotherapy sub-group contained one patient with resectable disease in each arm. When these patients were excluded, the overall survival benefit after 2 years additional follow-up was reduced and only just significant (*P*=0.049 log-rank test, HR=1.24 (1.00–1.53)). However, the result for the chemotherapy sub-group was largely unchanged (*P*=0.010 log rank test, HR=1.63 (1.12–2.37)).

The robustness of the survival benefit in the chemotherapy sub-group was explored further by Cox regression analyses to account for individual prognostic factor imbalance. None of the factors diminished the hazard ratio below 1.61, and the imbalance in prior surgical debulking increased the hazard ratio to 1.77. Furthermore, when divided in two halves on the basis of recruitment period, a survival trend in favour of marimastat is seen in both halves (HR=1.51 and HR=2.04).

Quality of life analyses using the EORTC-QLQC30 instrument were performed at baseline, 6 weeks, 12 weeks and at 3-monthly intervals thereafter. There was no statistically significant difference between placebo and marimastat in standardised area under the curve (AUC) (mean 57.9 *vs* 56.5, *P*=0.49) or change from baseline standardised AUC (mean −0.87 *vs* −3.82, *P*=0.18) over the first 12 weeks of the study (Wilcoxon rank-sum tests). Further analysis was prevented by a marked reduction in the number of patients remaining on study and able to complete the questionnaire.

### Safety and tolerability

Treatment related adverse events for marimastat appeared to be confined to the expected side effects of musculoskeletal pain and inflammation. In total 18 marimastat patients (10%) and two placebo patients (1%) withdrew from the study due to adverse events. Events of arthralgia, joint stiffness, limb pain, general pain, myalgia, peripheral oedema, dysphagia and fatigue were more common in the marimastat treatment arm (>5% absolute difference). The majority of these events were related to the musculoskeletal syndrome that has been described for marimastat and several other MMPIs. The condition is characterised by musculoskeletal pain and inflammation, most commonly originating in the upper shoulder girdle or hands. The condition tended to develop in the second and third months of treatment at 10 mg b.d. and in five cases led to contractures of the palm and fingers. A peripheral oedema of the hands was also sometimes seen in patients with these musculoskeletal side effects. The condition was managed by treatment interruption and many patients were able to resume treatment on the same or lower dose after a break of 2–3 weeks. Severe events could generally be avoided by early treatment interruption. A more general tiredness, recorded in a higher incidence of both fatigue and somnolence, may also be associated with marimastat treatment.

Events of abdominal pain, jaundice, weight loss, anaemia, and ascites were more common in the placebo treatment arm (>5% absolute difference). The increased incidence of anaemia was also evident in a 32% increase in transfusion requirement for the placebo arm over marimastat. This is consistent with the reduction in tumour haemorrhage observed endoscopically in the phase II study of marimastat in advanced gastric cancer, and with the intended therapeutic effect of marimastat.

The NCI-CTC graded adverse events (all causalities) are shown for both arms in Table 3

**Table 3:**
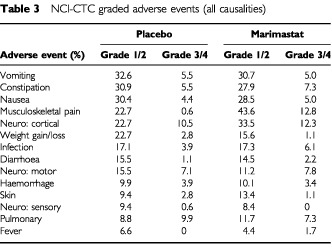
NCI-CTC graded adverse events (all causalities)

. Grades 3 and 4 musculoskeletal events were recorded in 12.8% of patients compared to 0.6% of placebo patients (*P*=0.001, χ^2^ test). A comparison of NCI-CTC graded laboratory abnormalities revealed an increased incidence of grade 3/4 low hemoglobin (<7.9 g dl^−1^) in the placebo arm (10.5% *vs* 2.8%, *P*=0.003, χ^2^ test) (Table 4

**Table 4:**
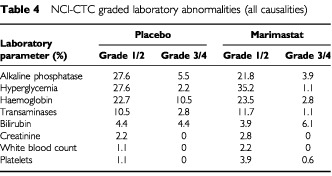
NCI-CTC graded laboratory abnormalities (all causalities)

). This was consistent with a higher transfusion requirement in placebo patients.

**Figure 4:**
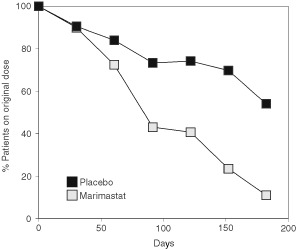
Patients taking original twice-daily dose as a percentage of those alive.

The significance of the musculoskeletal side effects of marimastat can be seen in Figure 4

. This graph shows the proportion of patients taking the prescribed dose of 10 mg b.d. marimastat or placebo without interruption over time, expressed as a percentage of patients alive. After 3 months only 43% of marimastat patients are still taking study treatment twice daily and this falls to 11% by 6 months. The corresponding values for the placebo arm were 73 and 50%. The decline in compliance in the placebo arm reflects both the background incidence of musculoskeletal conditions in cancer patients and also the deterioration in the patients' condition.

## DISCUSSION

The primary result of this randomised trial shows a modest survival benefit for marimastat treatment in the overall population of gastric cancer patients. The more interesting result, however, is seen in the sub-group of patients who had received prior chemotherapy. The survival benefit in this group, if true, is clinically significant with an absolute 2-year survival difference of 13%, and a 45% improvement in median survival. There are obvious concerns regarding the interpretation of a result from a sub-group even if, as in this case, it was pre-defined. For this reason the robustness of the result was explored further. Cox regression analysis indicated that imbalances of individual prognostic factors were unlikely to account for the survival difference. A survival benefit in favour of marimastat was also observed in both halves of the chemotherapy sub-group when divided arbitrarily on the basis of recruitment period. Finally, the result is not significantly reduced by the exclusion of the single patient in each arm of the sub-group who entered the study with potentially resectable disease (T1-2, N0-1).

It is important to note that the chemotherapy sub-group is a selected population, including as it does only those patients who had responded to chemotherapy or who had shown stable disease during treatment. The reason these patients responded to marimastat may be related to the fact that they responded in some way to the chemotherapy. Alternatively, the marimastat benefit may be related to the fact that this sub-group excludes those patients with more advanced or more rapidly progressive disease.

The safety and tolerability of marimastat was generally good, with only those events related to the musculoskeletal syndrome being significantly elevated above placebo. The musculoskeletal side effects clearly limit the duration of treatment for the majority of patients. However, the condition can be readily monitored and can be managed by treatment interruptions. In this study 21 placebo patients and 69 marimastat patients interrupted treatment, with a median duration of 14 days for both groups. Experience in this and other studies has shown that severe musculoskeletal side effects can usually be avoided by prompt treatment breaks. It is possible that improved treatment schedules and pre-medications could be employed to allow longer term dosing.

Early expectations for the use of MMP inhibitors in cancer treatment were high, but previous clinical trials with this new class of agent have been disappointing. Marimastat at a dose of 25 mg b.d. showed comparable 1-year survival to gemcitabine in patients with non-metastatic pancreatic cancer (Bramhall *et al*, 2001). A follow-on study of 10 mg b.d. marimastat in combination with gemcitabine, however, failed to show any evidence of survival benefit compared to gemcitabine alone. Studies of marimastat in patients with glioblastoma (British Biotech, 2000a), ovarian cancer (British Biotech, 2000b) and small cell lung cancer (British Biotech, 2001) also failed to show evidence of clinical benefit. Randomised, placebo-controlled studies with the MMP inhibitor AG3340 in patients with non-small cell lung cancer and prostatic cancer were stopped early on grounds of lack of efficacy (Agouron, 2000). Of more concern was the early termination of a randomised placebo-controlled study of the MMP inhibitor BAY12-9566 in patients with small cell lung cancer. Patients receiving BAY12-9566 showed a significantly poorer survival than placebo patients (Hughes, 1999). Reassuringly, an adverse effect on survival was not observed in the marimastat small cell lung cancer study (British Biotech, 2000c).

Against this background the current results are encouraging. This study of marimastat in patients with gastric cancer provides the first indication of a survival benefit for an MMP inhibitor. The reason that benefit is seen with this cancer and not others may be due to inadequate tissue concentrations of the drug. Biodistribution studies with ^14^C-marimastat in the rat have shown very high concentrations of marimastat in the stomach wall even after washing. By contrast the levels in lung were low (British Biotech – unpublished data).

In conclusion, these results support a possible role for marimastat as a maintenance treatment following a response or stable disease to chemotherapy. 5-FU based combination chemotherapy is gaining acceptance as the standard of care for gastric and gastro-oesophageal cancer patients with reasonable performance status, both in Europe and North America. In this setting, MMP inhibitor therapy may prove to be a valuable component of the anticancer armoury, and confirmatory trials of this novel agent are warranted.
